# The A148T variant of *CDKN2A* gene in bladder cancer

**DOI:** 10.1186/1897-4287-9-S2-A11

**Published:** 2011-06-01

**Authors:** E Borkowska, A Jedrzejczyk, A Kruk, M Traczyk, M Pietrusiński, P Marks, B Kaluzewski

**Affiliations:** 1 Medical University of Łodz, Chair of Clinical and Laboratory Genetics, Poland; 2 2nd Clinic of Urology Medical University of Łodz, Poland; 3 Department of Ecology and Vertebrate Zoology, University of Łodz, Poland; 4 Department of Urology, John Paul II Regional Hospital Belchatow, Poland

## Background

Cancer of the urinary bladder is a common malignant disease in Poland. Molecular genetic alterations play a key role in bladder cancer carcinogenesis. Understanding genetic events that lead to initiation and progression of bladder cancer remains an important challenge in urological research.

## Materials and methods

44 lesions were determined to be non-invasive tumors (pTa), whereas 36 tumors were invasive (pT1-T4). Tumor grade was noted low (G1) in 46 cases and high (G2-3) in 34 cases. DNA samples were evaluated for *CDKN2A* mutations with the use of MSSCP and sequencing method.

## Results

We found common polymorphic variants reported in the literature at codon 148 in exon 2 (Arg148Thr) and at nucleotides 500 and 540 in the 3’untranslated region which were not considered to be functional variants. We compared the obtained frequencies for the particular *CDKN2A* variants with the control group for the Polish population examined by Debniak and colleagues (Cancer Research 2005) and we found a significant difference in A148T polymorphism occurrence in the group of the bladder cancer patients (G test, table 2 ×2: N_BLADDER CANCER_ =80, N_CONTROL_=1210, G=10.214, p<0.01). The Nt 500c>g, Nt540c>t polymorphisms recorded in the group of the bladder cancer patients in our study is not different from those recorded in the control group.

## Conclusion

The A148T variant of *CDKN2A* gene seems to be associated with an increased risk of bladder cancer development.

